# Prediction of post-surgical seizure outcome in left mesial temporal lobe epilepsy^☆^

**DOI:** 10.1016/j.nicl.2013.06.010

**Published:** 2013-06-23

**Authors:** Delia-Lisa Feis, Jan-Christoph Schoene-Bake, Christian Elger, Jan Wagner, Marc Tittgemeyer, Bernd Weber

**Affiliations:** a Max Planck Institute for Neurological Research, Gleueler Straße 50, 50931 Cologne, Germany; b Dept. of Pediatrics, University of Freiburg Medical Center, Mathildenstraße 1, 79106 Freiburg, Germany; c Dept. of Epileptology, University of Bonn Medical Center, Sigmund-Freud-Str. 25, 53127 Bonn, Germany; d Life & Brain Center, Sigmund-Freud-Straße 25, 53127 Bonn, Germany

**Keywords:** Post-surgical seizure outcome prediction, Amygdalohippocampectomy, Support vector classification, Mesial temporal lobe epilepsy

## Abstract

Mesial temporal lobe epilepsy is the most common type of focal epilepsy and in its course often becomes refractory to anticonvulsant pharmacotherapy. A resection of the mesial temporal lobe structures is a promising option in these cases. However, approximately 30% of all patients remain with persistent seizures after surgery. In other words, reliable criteria for patients' outcome prediction are absent. To address this limitation, we investigated pre-surgical brain morphology of patients with unilateral left mesial temporal lobe epilepsy who underwent a selective amygdalohippocampectomy. Using support vector classification, we aimed to predict the post-surgical seizure outcome of each patient based on the pre-surgical T_1_-weighted structural brain images. Due to morphological gender differences and the evidence that men and women differ in onset, prevalence and symptomology in most neurological diseases, we investigated male and female patients separately. Thus, we benefitted from the capability to validate the reliability of our method in two independent samples. Notably, we were able to accurately predict the individual patients' outcome in the male (94% balanced accuracy) as well as in the female (96% balanced accuracy) group. In the male cohort relatively larger white matter volumes in the favorable as compared to the non-favorable outcome group were identified bilaterally in the cingulum bundle, fronto-occipital fasciculus and both caudate nuclei, whereas the left inferior longitudinal fasciculus showed relatively larger white matter volume in the non-favorable group. While relatively larger white matter volumes in the female cohort in the left inferior and right middle longitudinal fasciculus were associated with the favorable outcome, relatively larger white matter volumes in the non-favorable outcome group were identified bilaterally in the superior longitudinal fasciculi I and II. Here, we observed a clear lateralization and distinction of structures involved in the classification in men as compared to women with men exhibiting more alterations in the hemisphere contralateral to the seizure focus. In conclusion, individual post-surgical outcome predictions based on a single T_1_-weighted magnetic resonance image seem plausible and may thus support the routine pre-surgical workup of epilepsy patients.

## Highlights

• T_1_-weighted MRI and SVM predict outcome after amygdalohippocampectomy.
• Patients' individual outcome predictions reveal high sensitivity and specificity.
• Neuroanatomical regions outside the margins of resection were identified.
• A robust and patient-specific pre-surgical prediction of surgery outcome is presented.

## 1 Introduction

Epilepsy is a brain disorder characterized by episodes of disturbed brain activity (seizures) affecting the patient's attention and behavior (Engel, 2011). Mesial temporal lobe epilepsy (mTLE) is the most common type of focal epilepsy and in its course often becomes refractory to anticonvulsant pharmacotherapy (Engel, 1996, 2001; Faber et al., 2013; Focke et al., 2008; Schoene-Bake et al., 2009). In these cases, epilepsy surgery and resection of the mesial temporal lobe (mTL) structures after comprehensive pre-surgical diagnostics is a promising option that renders approximately 70% of the patients seizure free (Bien et al., 2013; Keller et al., 2007; Schulze-Bonhage, 2008; Wiebe et al., 2001). However, approximately 30% of all patients remain with persistent seizures after surgery. The cause of these persistent seizures often remains unclear, despite comprehensive ongoing research in this field (Bonilha et al., 2012; Thom et al., 2010). One possible reason might be the incomplete resection of the epileptogenic focus, however, numerous cases in which conventional magnetic resonance (MR) images indicate complete removal of the left mTL structures and no other possible epileptogenic lesion still exhibit post-surgical seizures. A voxel-based morphometry (VBM) study revealed that patients with poor surgical outcome had significantly reduced volumes of the ipsilateral posterior and contralateral medial temporal lobe compared to surgically remedied patients (Keller et al., 2007). Further VBM studies comparing patients and controls have demonstrated extrahippocampal changes of the ipsilateral temporal lobe and widespread structural alterations in white matter regions not restricted to the primarily affected temporal lobe (Bernasconi et al., 2005). New MR imaging techniques, such as diffusion MRI, have provided further evidence for extensive alterations of white matter fiber tracts (Faber et al., 2013; Focke et al., 2008; Schoene-Bake et al., 2009; Yogarajah et al., 2010). These extrahippocampal alterations in the cerebral white matter may also play a pivotal role in those cases with persistent post-surgical seizures. Thus, mTLE should rather be considered as a network disorder affecting brain structures both proximal to and distant from the seizure focus (McDonald et al., 2008).

Lately, an increasing number of studies have applied multivariate analysis methods such as support vector machines (SVMs) to predict the diagnostic status at a subject level of e.g. Alzheimer's disease (Klöppel et al., 2008), schizophrenia (Koutsouleris et al., 2011), the Turner syndrome (Marzelli et al., 2011), multiple sclerosis (Bendfeldt et al., 2012) or major depressive disorder (Mwangi et al., 2012). For more details the reader is referred to (Orrù et al., 2012). The SVM classification comprises of two stages. In the initial training phase, neuroimaging data from each subject and their corresponding diagnostic labels (e.g. favorable versus non-favorable outcome) are presented to the classifier. Thus, the system learns to categorize based on the given sample data. Neuroimaging data not previously used to train the classifier is then utilized to determine its diagnostic value and estimate the classification accuracy. A recent study proved the substantial contribution of SVMs for automated MR classification of patients with hippocampal sclerosis (Focke et al., 2012). Apart from the straightforward classification of mTLE patients from controls, the unambiguous determination of the lateralization is of great interest in pre-surgical evaluation. Perfect classification accuracy between left- and right-sided mTLE was demonstrated in the same study (Focke et al., 2012). Since it has been shown that left and right mTLE differ with respect to structural brain alterations not restricted to the temporal lobes as well as clinical characteristics, it seems useful to investigate these as distinct with respect to structural brain classification (Bernhardt et al., 2010).

To date, highly reliable criteria for patients' outcome prediction are still absent. Although hippocampal atrophy is recognized as a solid structural alteration predicting favorable post-surgical outcome (Bernhardt et al., 2010), some patients with clear left hippocampal sclerosis remain with residual seizures after surgery. Hence, the consideration of the hippocampus in isolation seems insufficient (Bernasconi et al., 2005). These patients motivated us to investigate additional morphometric markers for reliable outcome prediction. Post-surgical outcome classification based on a single T_1_-weighted MR image is of particular clinical interest, as these structural images are routine MR scans in pre-surgical evaluation. We hypothesized that we can distinguish between those patients with good and poor surgical outcomes using pre-surgical high-resolution MR images.

Considering the aspects of (i) the evidence of a considerable reorganization of white matter (WM) connectivity in the speech dominant (usually left) hemisphere (Powell et al., 2007), (ii) a more widespread atrophic distribution (Bernhardt et al., 2010) and (iii) more extensive alterations in left mTLE (Focke et al., 2008), we decided to focus on the white matter of mTLE patients with unilateral left-sided mTLE. Given morphological sex differences in the human brain (Feis et al., 2013) and the fact that most neurological illnesses differ in onset, prevalence and symptomatology between females and males (Giedd et al., 2012), we decided to split the group into a male and a female cohort. Thus, we additionally benefit from the advantage to investigate the capability of the SVM for post-surgical outcome prediction in two independent samples. We were able to precisely distinguish between those patients with good versus poor surgical outcome using their pre-surgical high-resolution MR images. Our method further identified the spatial organization of neuroanatomical structures associated with the specific outcome group.

## 2 Materials and methods

### 2.1 Subjects

Inclusion criteria for this retrospective analysis were: (i) unilateral left mesial temporal lobe epilepsy according to pre-surgical workup and unilateral selective amygdalohippocampectomy, (ii) no lesion other than left sided hippocampal sclerosis on the pre-surgical MRI (iii) no peri- or post-surgical complications, and (iv) post-surgical outcome rating at least one year after surgery. The ILAE outcome classification was used as post-surgical outcome rating (Wieser et al., 2001). Here, ILAE classes 1 and 2 were considered a favorable outcome (FO) and the remaining classes (3–6) as a non-favorable outcome (Non-FO).

According to these criteria, 49 patients (19 males, mean age ± SD: 41 ± 13 years) who were operated at our hospital between 2007 and 2011 were included in the study. All patients underwent high-resolution structural 3 Tesla-MRI as part of our regular pre-surgical workup that in all cases included neuropsychological tests, interictal and ictal video electroencephalography (EEG) monitoring. Forty-four patients showed a unilateral left-sided hippocampal sclerosis on their pre-surgical MRI (16 males, 28 females) which was histologically confirmed in all cases after surgery. The remaining five non-lesional patients underwent invasive pre-surgical diagnostic where bilateral intrahippocampal depth electrodes were implanted. A left-sided mesial temporal seizure focus was diagnosed in all of these five patients. An overview of demographic characteristics and clinical information for the two patient groups is provided in Table 1. Seventeen of the patients had a history of childhood febrile seizures. Inline Supplementary Tables S1 and S2 provide further detailed information of the male and female cohorts, respectively. Because of previously described differences between males and females detected by SVM analysis, separate analyses were performed for males and females. Since we consequently use two independent samples, we were able to compare the capability of the SVM for post-surgical outcome prediction. Both cohorts revealed no significant difference between the FO and Non-FO groups with respect to age at MRI (p = 0.67 for males and p = 0.98 for females; Mann–Whitney *U* test). All subjects gave written informed consent, and the ethics committee of the University of Bonn approved the study.

According to these criteria, 49 patients (19 males, mean age ± SD: 41 ± 13 years) who were operated at our hospital between 2007 and 2011 were included in the study. All patients underwent high-resolution structural 3 Tesla-MRI as part of our regular pre-surgical workup that in all cases included neuropsychological tests, interictal and ictal video electroencephalography (EEG) monitoring. Forty-four patients showed a unilateral left-sided hippocampal sclerosis on their pre-surgical MRI (16 males, 28 females) which was histologically confirmed in all cases after surgery. The remaining five non-lesional patients underwent invasive pre-surgical diagnostic where bilateral intrahippocampal depth electrodes were implanted. A left-sided mesial temporal seizure focus was diagnosed in all of these five patients. An overview of demographic characteristics and clinical information for the two patient groups is provided in Table 1. Seventeen of the patients had a history of childhood febrile seizures. Inline Supplementary Tables S1 and S2 provide further detailed information of the male and female cohorts, respectively. Because of previously described differences between males and females detected by SVM analysis, separate analyses were performed for males and females. Since we consequently use two independent samples, we were able to compare the capability of the SVM for post-surgical outcome prediction. Both cohorts revealed no significant difference between the FO and Non-FO groups with respect to age at MRI (p = 0.67 for males and p = 0.98 for females; Mann–Whitney *U* test). All subjects gave written informed consent, and the ethics committee of the University of Bonn approved the study.

Inline Supplementary Table S1

**Table S1:** Individual clinical details of all male patients. *Abbreviations*: MRI = magnetic resonance imaging; SPS = simple partial seizure; CPS = complex partial seizure; SGTCS = secondarily generalized tonic clonic seizure; ILAE = international league against epilepsy; AED = antiepileptic drugs; FU = follow-up; ZNS = zonisamide; LEV = levetiracetam; PGB = pregabalin; LTG = lamotrigine; OXC = oxcarbazepine; LCM = lacosamide; CLB = clobazam; CBZ = carbamazepine; PRM = primidone; ESL = eslicarbazepine; VPA = valproic acid; LRZ = lorazepam.

ID	Age at MRI	Age at onset	febrile seizures	Age at surgery	Pre-surgical seizure type	Seizure frequency per month	Duration of follow-up in months	Last available outcome (ILAE)	AED at surgery	AED at last FU
M01	56	24	1	56	CPS, SGTCS	3	24	1	ZNS	PGB
M02	51	33	0	51	CPS, SGTCS	3	24	1	LEV, LTG	LEV, LTG
M03	53	1	0	52	SPS, CPS, SGTCS	6	18	1	LTG. LEV	LEV
M04	32	13	1	32	SPS, CPS	4	16	2	LEV, LTG	LEV, LTG
M05	41	5	0	42	SPS, SGTCS	3	30	1	LEV, OXC	LEV, OXC
M06	57	34	0	57	SPS, CPS, SGTCS	20	13	1	OXC	LTG
M07	50	26	0	50	SPS, CPS	5	26	1	LEV, LTG	LEV, LTG
M08	22	1	0	23	SPS, CPC	12	23	1	LEV, LCM	LEV, LCM
M09	31	3	1	31	SPS, CPS	1	19	2	LTG	LTG
M10	27	18	1	28	SPS, SGTCS	5	12	1	LEV, LTG	LEV, LTG
M11	37	35	0	37	CPS, SGTCS	10	19	1	LEV, LTG	LEV, LTG
M12	67	46	0	67	CPS, SGTCS	40	12	4	LEV, LTG, OXC	LEV, LTG, OXC
M13	47	5	1	47	CPC, SGTCS	4	45	3	LEV, LTG, CLB	LTG, LEV
M14	21	18	0	21	CPS	4	12	3	LTG, LEV	LTG, LEV
M15	49	15	1	50	CPS	1	30	3	LEV, LTG, CLB	LEV, LTG
M16	46	3	1	46	SPS, CPS	8	28	4	LEV	LEV
M17	44	11	0	45	SPS, CPS	3	12	4	OXC	CBZ
M18	51	5	0	51	SPS, CPS	5	25	4	LTG	LTG, OXC
M19	51	42	1	52	SPS, CPS, SGTCS	5	20	3	LEV

Inline Supplementary Table S1

Inline Supplementary Table S2

**Table S2:** Individual clinical details of all female patients. *Abbreviations*: MRI = magnetic resonance imaging; SPS = simple partial seizure; CPS = complex partial seizure; SGTCS = secondarily generalized tonic clonic seizure; ILAE = international league against epilepsy; AED = antiepileptic drugs; FU = follow-up; ZNS = zonisamide; LEV = levetiracetam; PGB = pregabalin; LTG = lamotrigine; OXC = oxcarbazepine; LCM = lacosamide; CLB = clobazam; CBZ = carbamazepine; PRM = primidone; ESL = eslicarbazepine; VPA = valproic acid; LRZ = lorazepam.

ID	Age at MRI	Age at onset	Febrile seizures	Age at surgery	Pre-surgical seizure type	Seizure frequency per month	Duration of follow-up in months	Last available outcome (ILAE)	AED at surgery	AED at last FU
F01	35	2	0	36	SPS, CPS	4	38	1	LEV	LEV
F02	64	14	0	64	CPS	3	48	1	LTG. LEV	PGB. LRZ
F03	22	0	0	23	CPS	30	25	1	LEV, CLB, LTG	LTG
F04	49	2	1	49	SPS, CPS	1	39	1	PRM, LEV	PRM, LEV
F05	39	29	0	39	CPS, SGTCS	3	36	1	LEV, LTG	LTG
F06	44	15	0	44	SPS, CPS	4	13	1	LEV	LEV, LCM
F07	45	21	0	46	CPS	15	24	1	LEV, OXC	LEV, OXC
F08	42	9	0	42	SPS, CPS	60	28	1	LTG, LEV	LEV, LTG
F09	27	13	1	27	SPS, CPS	2	12	1	LTG	LTG
F10	30	18	1	31	CPS, SGTCS	5	12	1	LEV, OXC	LEV, OXC
F11	33	13	0	34	SPS, CPS, SGTCS	2	24	2	LEV, LTG	LEV, LTG
F12	45	3	1	46	CPS	3	12	1	LEV	LEV
F13	48	30	0	49	SPS, CPS, SGTCS	15	12	1	LTG, PGB	LTG, PGB
F14	22	13	0	22	SPS, CPS	4	29	1	LTG, LCM	LTG
F15	32	29	0	32	SPS	8	18	2	LEV, LCM	LEV, LCM
F16	54	14	0	54	SPS, CPS, SGTCS	1	12	1	LEV, LTG	LEV, LTG
F17	39	36	0	40	CPS	4	17	2	LTG	LTG
F18	29	15	1	29	CPS	6	12	2	LEV	LEV
F19	24	6	0	26	CPS	1	38	3	LTG	LTG
F20	25	11	1	25	CPS, SGTCS	5	54	3	LTG, LEV	LTG
F21	33	32	0	33	SPS, CPS, SGTCS	10	52	3	LEV, LTG, LRZ	LTG, LEV, LCM, LRZ
F22	32	6	1	32	SPS, CPS, SGTCS	7	34	4	LEV, CLB	LCM, VPA, LTG, ZNS
F23	56	33	0	57	SPS, CPS	1	16	4	LTG	LTG
F24	43	1	0	43	CPS, SGTCS	2	22	4	CBZ, LTG	LTG, ESL
F25	23	13	0	24	CPS, SGTCS	1	23	4	LTG	LTG
F26	26	9	0	26	SPS, CPS	4	12	3	OXC	OXC, LEV
F27	50	15	1	51	CPS, SGTCS	6	23	4	CBZ, CLB	CBZ
F28	70	34	0	72	CPS	6	12	3	LEV, LCM	LTG
F29	41	1	1	41	CPS, SGTCS	3	16	4	CBZ, LTG	LTG, OXC
F30	62	21	0	63	SPS, CPS	2	12	3	LTG	LTG

Inline Supplementary Table S2

Inline Supplementary Tables S1 and S2 can be found online at http://dx.doi.org/10.1016/j.nicl.2013.06.010.

### 2.2 MRI data acquisition

High-resolution T_1_-weighted images were acquired using a 3 Tesla Siemens Magnetom Trio scanner (8-channel array head coil) with a whole-brain field of view (T_1_-weighted: MPRAGE; TR = 1300 ms, TI = 650 ms, TE = 3.97 ms, resolution = 1 ×  1 ×  1 mm^3^, flip angle = 10°, 160 sagittal slices).

### 2.3 Image preprocessing

The VBM8 toolbox (http://dbm.neuro.uni-jena.de/vbm/) was used to preprocess the acquired T_1_-weighted images (Feis et al., 2013). Initially, these images were corrected for bias-field inhomogeneities and registered nonlinearly to a template derived from 550 healthy volunteers of the IXI database (http://www.brain-development.org/). Anatomical segmentation into gray matter (GM) and white matter (WM) was attained using a *maximum a posteriori* (MAP) technique (Rajapakse et al., 1997), accounting for partial volume effects (Tohka et al., 2004) and applying denoising methods such as a hidden Markov random field model (Cuadra et al., 2005). Finally, the WM segments were smoothed using an isotropic Gaussian kernel of 3 mm full-width-half-maximum (Jones and Cercignani, 2010).

### 2.4 Classification

In order to discriminate between FO and Non-FO brains on the basis of WM segments, we used a supervised, multivariate classification method called support vector machine (SVM, as implemented by (Chang and Lin, 2011)). In a binary classification, an SVM learns to separate two groups given labeled example training data. Here, the training set {*X*_*i*_,*y*_*i*_}_*i* = 1_^*N*^ for *N* subjects is represented by a training sample *X_i_* and its diagnostic label *y_i_* (favorable versus non-favorable outcome). In this context, each WM segment of the T_1_-weighted MR image is treated as a single point in a high dimensional space. The number of voxels in each WM segment (*n*) indicates the number of dimensions, thus, coordinates in this space are determined by the intensity values at each voxel. The objective was to train a model that accurately predicts *y* of previously unseen imaging data *X* (testing stage). For this purpose, during training stage *X* is not provided. This training concept is called ‘leave-one-subject-out’ cross-validation (Lemm et al., 2011) as all but one patient are used to create the SVM model. In the training step of the SVM a decision function or hyperplane $f:R^{n}\to \left\{-1,1\right\}$ was identified that assigns the brain imaging data to either the negative or positive class. In our study, the surgically remedied patients form the positive class; the patients rendered with persistent post-surgical seizures form the negative class.

SVM is based on the principle of ‘structural risk minimization’ (Vapnik, 1998), which aims to find an optimal hyperplane that maximizes the distance between the two classes (favorable versus non-favorable outcome), simultaneously minimizing data misclassification. The individual subjects closest to the optimal hyperplane constitute it and are termed ‘support vectors’. Thus, the closer an individual is to the identified hyperplane, the more ambiguous it is. Conversely, rather distant individuals are more distinct.

**Fig. 1:**
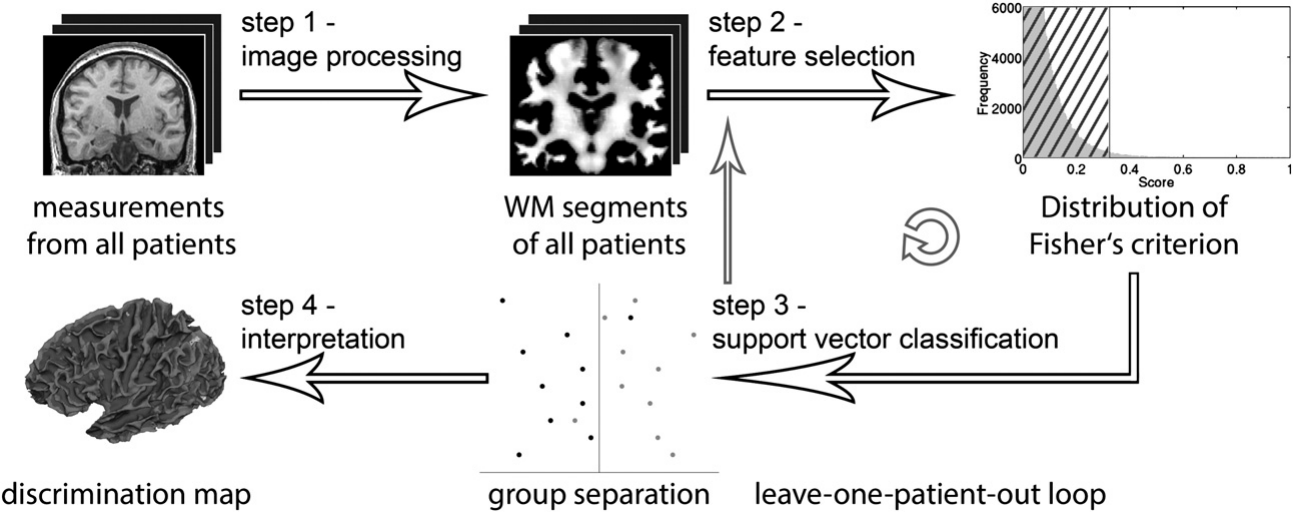
Flow diagram illustrating our method. After image processing (step 1), the feature selection (step 2) as well as the support vector classification (step 3) are repeated in a leave-one-patient-out manner until all patients have been left out once. An overall balanced accuracy can be computed from each repetition (step 3). In conclusion, we can interpret the spatial deployment of weights in the original anatomical space (step 4).

An SVM model requires two parameters: a ‘kernel’ and a ‘regularization’ parameter. In our study, we use a linear kernel and the regularization parameter was identified using a ‘grid search’ method within a leave-one-subject-out cross-validation procedure during training phase. Hence, we form three groups of patients to validate our method: (i) one patient is ‘left out’ as test subject, (ii) another patient is omitted as validation subject, and (iii) the remaining patients (N − 2) are used as training set to create the SVM model (Feis et al., 2013). This procedure is called ‘nested-leave-one-subject-out’ cross-validation (Lemm et al., 2011). The regularization parameter (chosen within the inner cross-validation) allows defining a maximal margin between the two classes and at the same time minimizing misclassification. While the inner cross-validation is used for model selection, the outer cross-validation ensures an unbiased model evaluation. Hence, the leave-one-subject-out cross-validation scheme ensures generalization of the SVM model. In other words, the model is able to correctly assign previously unseen data *X* to the appropriate class *y* (Fig. 1). Due to the use of a linear kernel, we are able to extract a weight vector reflecting the importance of each voxel for classification. This makes it possible to assess the spatial deployment of weights in the original anatomical space. The resulting maps are called ‘discrimination maps’. Further technical details can be found in Vapnik (1998).

The prediction performance of the SVM was evaluated using a 2 × 2 ‘confusion matrix’, obtained from the classifier testing step, and used to calculate sensitivity, specificity, positive predictive value, false and true positive rate as well as balanced posterior accuracy (Brodersen et al., 2010) with their 95% credible interval. Additionally, a receiver operating characteristic (ROC) curve and its area under ROC curve were generated.

Typically, a WM segment of a T_1_-weighted image contains more voxels than numbers of subjects in our study and it includes ‘noise’. Thus, we decided to preselect the most important brain regions using feature selection to ensure accurate predictions. Notably, the feature selection is only performed on the training set to ensure a model selection independent of the omitted test subject. Once determined, the same subset of important brain regions in unseen data *X* is used to predict a label *y*. Here, we used a ranker method called Fisher's criterion (Furey et al., 2000). This score reflects the squared distance between the class means $\hat{\mu }\left(\cdot \right)$ in relation to the intra-class standard deviations $\hat{\sigma }\left(\cdot \right):f_{v}\left(X\right)={\left(\hat{\mu }\left(X^{+}\right)-\hat{\mu }\left(X^{-}\right)\right)}^{2}/\hat{\sigma }\left(X^{+}\right)+\hat{\sigma }\left(X^{-}\right)$, whereby *X*^+^ denotes the FO patients or positively labeled class; consequently, *X*^−^ depicts the Non-FO patients or negatively labeled class. Subsequently, we ranked the scores according to how informative each one is with respect to discriminating the two groups (Müller et al., 2001). Thus, during the inner cross-validation only the highest ranked scores were automatically selected via grid search to enter the analysis (Furey et al., 2000). The most discriminative features are not restricted to one specific brain region, but as can be seen in the discrimination maps are rather spatially distributed.

## 3 Results

Table 1 summarizes sociodemographic and clinical details of the patients. The patients were separated into gender-specific groups. We found no significant differences with respect to any clinical characteristic in the male or female cohort. The two groups (favorable versus non-favorable outcome) were compared using a Mann–Whitney *U* test in the male and female cohorts. Additionally, the number of patients who had febrile seizures during their childhood did not differ in the male (p = 0.66; Fisher's exact test) or female cohort (p = 0.71; Fisher's exact test) between the two groups (FO versus Non-FO). In order to further validate the difference between both cohorts, we used a four group Kruskal–Wallis rank sum test for most clinical characteristics and the Fisher's exact test for the history of febrile seizures. No differences between the cohorts were observed. We examined the male cohort first and then subsequently used the female sample to replicate our findings.

### 3.1 Patients' individual outcome predictions indicate excellent performance

**Fig. 2:**
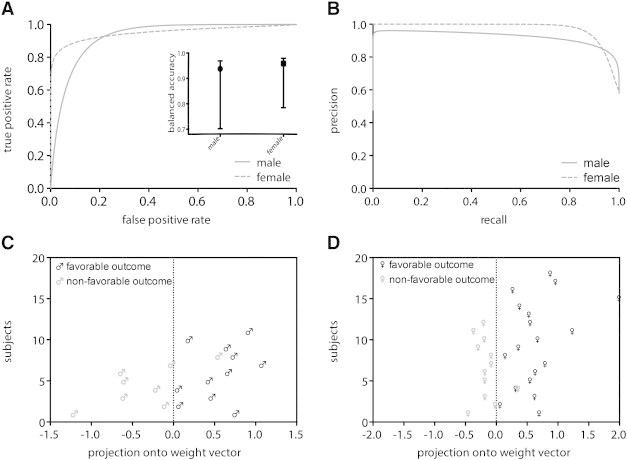
Classification results. (A) Receiver operating characteristic curves of the male and female cohorts. The balanced classification accuracies with 95% credible intervals are included. (B) Precision recall curves of the male and female cohorts. (C) Best male classification accuracy (94%) provided utilizing T_1_-weighted white matter segments. (D) Best female separation (96%) achieved by the classifier utilizing T_1_-weighted white matter segments.

We found the best classification performance of male FO versus Non-FO brains using 310 voxels of the T_1_-weighted WM segments. Totally, a balanced accuracy of 94% (with a 95% credible interval of 70% to 97%, Fig. 2B) with a sensitivity of 100% and a specificity of 88% was achieved. To visualize the separability of the male patients, we projected the data features onto the weight vector of the SVM (Fig. 2C). In other words, all but one male patient were correctly predicted using our framework. Patient specific decision values of the classifier are provided in Inline Supplementary Table S3. The ROC curve yields an area under curve (AUC) totaling 0.93 with an F-measure of 0.96 (Fig. 2A). Overall, these statistical results indicate excellent performance across three performance metrics: balanced posterior accuracy, area under curve and F-measure.

We found the best classification performance of male FO versus Non-FO brains using 310 voxels of the T_1_-weighted WM segments. Totally, a balanced accuracy of 94% (with a 95% credible interval of 70% to 97%, Fig. 2B) with a sensitivity of 100% and a specificity of 88% was achieved. To visualize the separability of the male patients, we projected the data features onto the weight vector of the SVM (Fig. 2C). In other words, all but one male patient were correctly predicted using our framework. Patient specific decision values of the classifier are provided in Inline Supplementary Table S3. The ROC curve yields an area under curve (AUC) totaling 0.93 with an F-measure of 0.96 (Fig. 2A). Overall, these statistical results indicate excellent performance across three performance metrics: balanced posterior accuracy, area under curve and F-measure.

Inline Supplementary Table S3

**Table S3:** Prediction of male patients with their individual subject results.

Subject number	Actual diagnostic/outcome label 1—favorable − 1—non-favorable	SVM predicted label^⁎^
M01	1	0.75914
M02	1	0.075861
M03	1	0.4679
M04	1	0.059285
M05	1	0.43343
M06	1	0.67053
M07	1	1.0851
M08	1	0.73569
M09	1	0.65359
M10	1	0.19013
M11	1	0.92097
M12	− 1	− 1.2076
M13	− 1	− 0.097875
M14	− 1	− 0.60293
M15	− 1	− 0.20462
M16	− 1	− 0.59343
M17	− 1	− 0.63184
M18	− 1	− 0.023385
M19	− 1	0.55178

Predicted label designated as ^⁎^> 0 = favorable or < 0 = non-favorable outcome. The further apart a prediction is from 0, the stronger is the evidence of that patient being in either of the groups.

Inline Supplementary Table S3

Inline Supplementary Table S3 can be found online at http://dx.doi.org/10.1016/j.nicl.2013.06.010.

In the female cohort the best classification was reached using 360 voxels of the T_1_-weighted WM segments. Here, a balanced classification accuracy of 96% (with a 95% credible interval of 78% to 98%, Fig. 2B) with a sensitivity of 100% and a specificity of 92% was attained. We also projected the data features of the female classification onto its weight vector to highlight the considerable separability (Fig. 2D). Numerically speaking, only one female patient was misclassified. Patient specific decision values of the female classifier are provided in Inline Supplementary Table S4. The ROC curve is totaling an AUC of 0.95 with an F-measure of 0.97 (Fig. 2A). This demonstrates once more an excellent and significantly above chance classification performance to distinguish between female patients with a good versus poor surgical outcome. For a numerical summary of these results, see Table 2.

In the female cohort the best classification was reached using 360 voxels of the T_1_-weighted WM segments. Here, a balanced classification accuracy of 96% (with a 95% credible interval of 78% to 98%, Fig. 2B) with a sensitivity of 100% and a specificity of 92% was attained. We also projected the data features of the female classification onto its weight vector to highlight the considerable separability (Fig. 2D). Numerically speaking, only one female patient was misclassified. Patient specific decision values of the female classifier are provided in Inline Supplementary Table S4. The ROC curve is totaling an AUC of 0.95 with an F-measure of 0.97 (Fig. 2A). This demonstrates once more an excellent and significantly above chance classification performance to distinguish between female patients with a good versus poor surgical outcome. For a numerical summary of these results, see Table 2.

Inline Supplementary Table S4

**Table S4:** Prediction of female patients with their individual subject results.

Subject number	Actual diagnostic/outcome label 1—favorable − 1—non-favorable	SVM predicted label^⁎^
F01	1	0.69374
F02	1	0.057686
F03	1	0.61948
F04	1	0.31212
F05	1	0.54516
F06	1	0.62866
F07	1	0.78818
F08	1	0.14086
F09	1	0.3572
F10	1	0.66793
F11	1	1.2308
F12	1	0.54714
F13	1	0.52743
F14	1	0.3758
F15	1	1.9909
F16	1	0.26292
F17	1	0.95757
F18	1	0.87285
F19	1	− 0.46047
F20	− 1	− 0.020884
F21	− 1	− 0.18794
F22	− 1	0.35073
F23	− 1	− 0.1866
F24	− 1	− 0.18786
F25	− 1	− 0.085392
F26	− 1	− 0.086466
F27	− 1	− 0.29476
F28	− 1	− 0.19536
F29	− 1	− 0.3722
F30	− 1	− 0.21038

Predicted label designated as ^⁎^> 0 = favorable or < 0 = non-favorable outcome. The further apart a prediction is from 0, the stronger is the evidence of that patient being in either of the groups.

Inline Supplementary Table S4

Inline Supplementary Table S4 can be found online at http://dx.doi.org/10.1016/j.nicl.2013.06.010.

### 3.2 Neuroanatomical regions outside the margins of resection identified

**Fig. 3:**
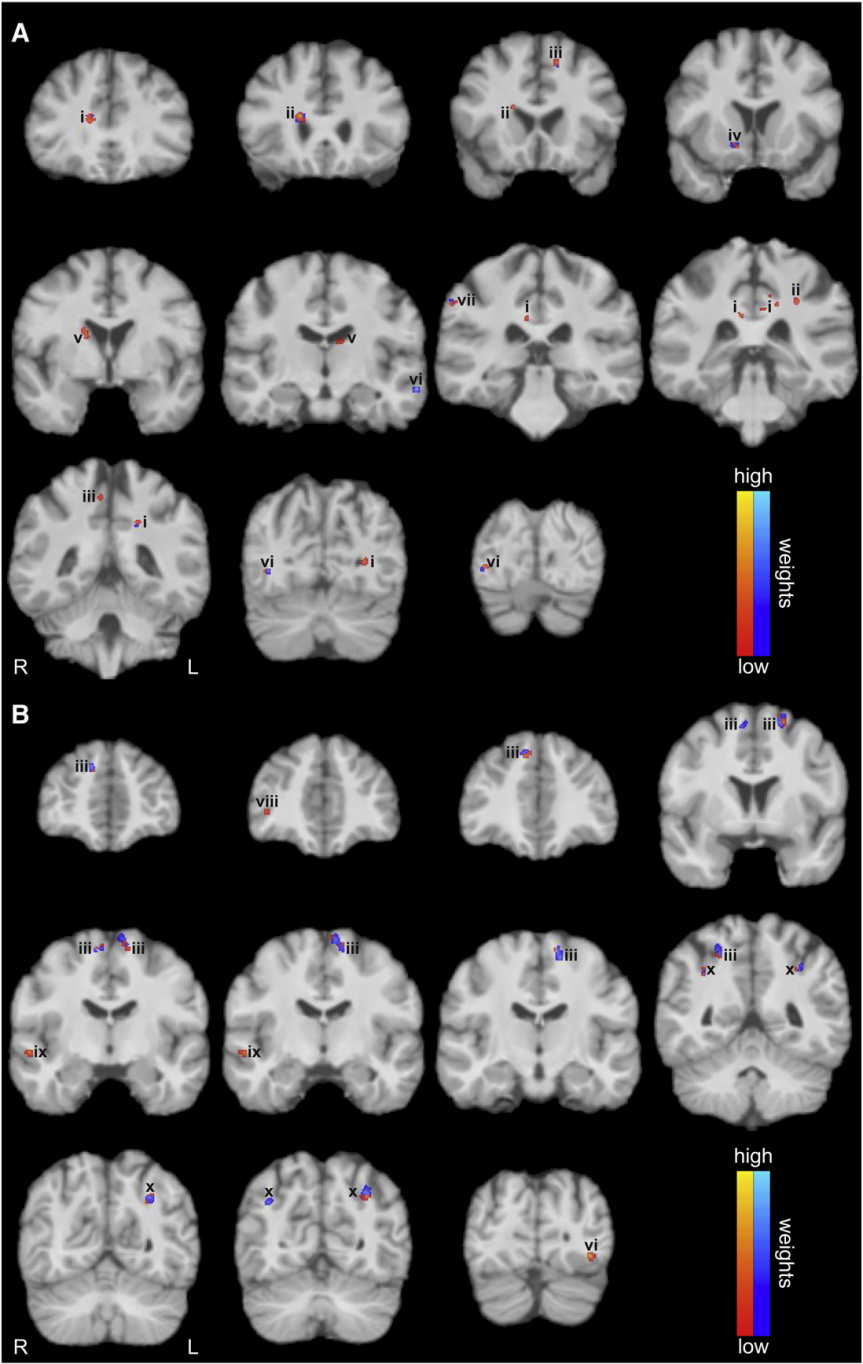
Discrimination maps derived from the classification of white matter segments of T_1_-weighted images in the (A) male and (B) female cohorts. Results are superimposed onto a T_1_-weighted image of an individual study brain. The resulting regions comprise spatially contiguous patterns of relatively larger WM volume (positive weight vector; red color scale) or relatively smaller WM volume (negative weight vector; blue color scale) in the favorable as compared to the non-favorable group, respectively. Labels: (i) cingulum bundle, (ii) fronto-occipital fasciculus, (iii) superior longitudinal fasciculus I, (iv) internal capsule, (v) caudate nucleus, (vi) inferior longitudinal fasciculus, (vii) superior longitudinal fasciculus III, (viii) extreme capsule, (ix) middle longitudinal fasciculus, and (x) superior longitudinal fasciculus II. L and R indicate the left and right hemispheres, respectively.

**Fig. 4:**
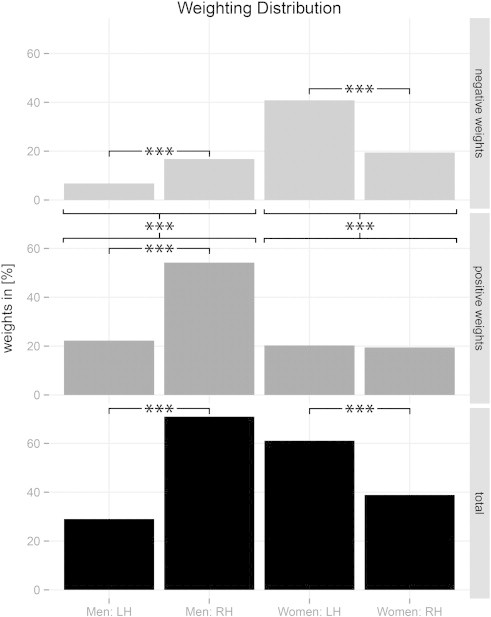
Weighting distribution found in T_1_-weighted white matter segments in the male and female cohorts. Besides the distinction of left (LH) and right (RH) hemispheres, total weighting is split into its positive and negative proportions, respectively. In the male and female cohorts the lateralization of the total weighting is statistically significant (*** = highly significant, p < 0.001; Chi-square test).

In order to identify the spatial organization of neuroanatomical structures associated with the specific outcome group, we considered the discrimination maps that are based on the weights attributed to each voxel by the SVM (Fig. 3). Regions showing disparities between the favorable and non-favorable surgery outcomes were found in the male (Fig. 3A) as well as in the female cohort (Fig. 3B). Interestingly, while the weighting distribution is significantly lateralized towards the right hemisphere in men (p < 0.001; Chi-square test), the women show a significant lateralization towards the left hemisphere (p < 0.001; Chi-square test; Fig. 4). The positive and negative weightings were subsequently analyzed in relation to their total weighting amount. Both cohorts show a significant difference between their positive and negative weightings, though they reveal a converse behavior. While the men primarily exhibit positive features that contribute to a favorable surgery outcome (p < 0.001; Chi-square test), the women possess significantly more negative weights contributing to a non-favorable surgery outcome (p < 0.001; Chi-square test; Figs. 3, 4). Similarly to their total weight proportions, the male patients indicate a strong and statistically significant lateralization towards the right hemisphere (which is contralateral to their seizure focus) in the positive as well as in the negative weights (p < 0.001; Chi-square test). However, the female patients only show a difference in hemisphere lateralization in negative weights (p < 0.001; Chi-square test). The positive weights are uniformly distributed along both hemispheres (Fig. 4).

In the male cohort relatively larger WM volumes in favorable as compared with the non-favorable outcome group (positive weight vector; red color scale) were found bilaterally in the cingulum bundle (CB), the fronto-occipital fasciculus (FOF), the superior longitudinal fasciculus (SLF) I, the caudate nuclei and in the inferior longitudinal fasciculus (ILF). Here, the disparity found in the left ILF reveals a relatively larger WM volume in the non-favorable as compared with the favorable outcome group (negative weight vector; blue color scale). Relatively larger WM volume in the favorable as compared to the non-favorable outcome group is further indicated by the disparity found in the right SLF III. In addition, differences in the internal capsule (ICA) occurred only in the right hemisphere.

Conversely to the weighting distribution in the male cohort, women tended to show overall more regions with relatively larger WM volumes in the non-favorable as compared with the favorable outcome (negative weight vector; blue color scale). Bilateral differences between the two categories were found in the SLF I as well as in the SLF II. The only disparities indicating relatively larger WM volumes in the favorable as compared with the non-favorable outcome group (positive weight vector; red color scale) were found in the right extreme capsule, the right middle longitudinal fasciculus (MdLF) and the left ILF. Briefly, both cohorts exhibit neuroanatomical regions outside the margins of resection as well as in the contralateral hemisphere attributed to both outcome types.

### 3.3 No correlations between support vector weighting and clinical characteristics found

**Fig. S1:**
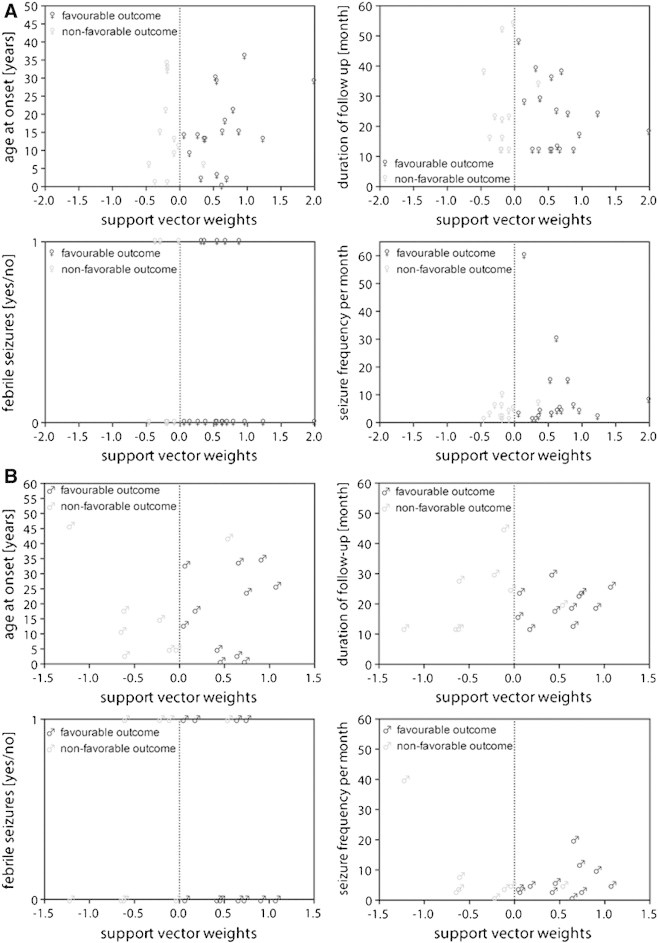
Scatter plots illustrating the relationship between the support vector weighting and clinical characteristics in the (A) male and (B) female cohorts. Clinical characteristics are (i) age at onset given in years, (ii) duration of follow-up given in months, (iii) the history of febrile seizures (yes = 1 or no = 0) and (iv) the seizure frequency per month.

We analyzed correlations between the resulting support vector weighting and four clinical characteristics, namely (i) the age at onset given in years, (ii) the duration of follow-up given in months, (iii) the history of febrile seizures during childhood and (iv) their seizure frequency per month. As was expected, no correlations for the male as well as the female cohorts were found (see Inline Supplementary Fig. S1).

We analyzed correlations between the resulting support vector weighting and four clinical characteristics, namely (i) the age at onset given in years, (ii) the duration of follow-up given in months, (iii) the history of febrile seizures during childhood and (iv) their seizure frequency per month. As was expected, no correlations for the male as well as the female cohorts were found (see Inline Supplementary Fig. S1).

Inline Supplementary Figure S1

Inline Supplementary Fig. S1 can be found online at http://dx.doi.org/10.1016/j.nicl.2013.06.010.

### 3.4 No differences found in clinical characteristics of support vector machine support and non-support categorized patients

**Fig. S2:**
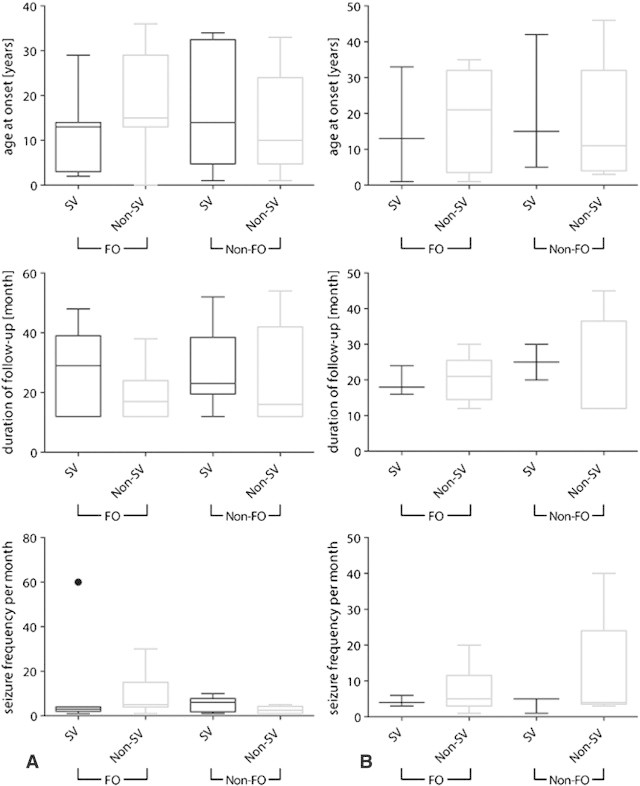
Boxplots demonstrating the clinical characteristics of those patients in both groups (favorable outcome = FO and non-favorable outcome = Non-FO) categorized as providing support vector image data (SV) versus those providing non-support vector data (Non-SV) for the (column A) male and (column B) female cohorts. Clinical characteristics used here are (i) age at onset given in years, (ii) duration of follow-up given in months, and (iii) the seizure frequency per month. No significant differences between groups were observed (Kruskal–Wallis rank sum test).

After classification we were able to identify the patients of both groups (FO vs Non-FO) who provided relevant imaging data for the classifier. Thus, we evaluated the hypothesis of no difference between the patients contributing ‘support vector’ (SV) data and Non-SV patients of the FO and Non-FO groups (see Inline Supplementary Fig. S2). We found no significant differences in the male as well as the female cohorts.

After classification we were able to identify the patients of both groups (FO vs Non-FO) who provided relevant imaging data for the classifier. Thus, we evaluated the hypothesis of no difference between the patients contributing ‘support vector’ (SV) data and Non-SV patients of the FO and Non-FO groups (see Inline Supplementary Fig. S2). We found no significant differences in the male as well as the female cohorts.

Inline Supplementary Figure S2

Inline Supplementary Fig. S2 can be found online at http://dx.doi.org/10.1016/j.nicl.2013.06.010.

## 4 Discussion

To date, although the presence of an atrophic hippocampus is often recognized as an important diagnostic factor for a good surgical outcome (Focke et al., 2012), 30% of all patients remain with persistent seizures after surgery (Keller et al., 2007). Patients with a non-lesional mTLE have a chance of surgical success below 50% (Bien et al., 2009; Téllez-Zenteno et al., 2010). Thus, the clinical diagnostic of mTLE patients is so far lacking robust criteria for solid surgery outcome prediction. Our results clearly indicate the feasibility to precisely predict the outcome after an amygdalohippocampectomy for left mTLE patients using pre-surgical T_1_-weighted MR scans and support vector classification. Due to morphological sex differences in the human brain (Feis et al., 2013) and the distinctions in onset, prevalence and symptomatology of most neurological illnesses between men and women (Giedd et al., 2012), we analyzed the male and female patients in separate cohorts. Strikingly distinct pattern of brain structures contributing to the individual outcome were observed in men as compared to women. Hence, pointing to the importance of a separate investigation when predicting the patients' surgical outcome. Our data further extend the literature by providing evidence indicating that both surgery outcome types are associated with different structural WM alterations outside the margins of resection as well as in the contralateral hemisphere.

### 4.1 Patients' individual outcome predictions revealed high sensitivity and specificity

The approach described here was initially used for the prediction of a favorable versus non-favorable surgery outcome in a small group of male patients. We further analyzed the capability of our framework using a slightly larger set of female patients. The multivariate pattern analyses revealed convincing prediction accuracies in the two independent cohorts (Fig. 2). The male and female patients achieved a balanced classification accuracy of 94% and 96% with an area under the ROC curve of 0.93 and 0.95, respectively (Table 2). These results indicate an excellent performance across both performance metrics: balanced prediction accuracy and area under the ROC curve. Considering these robust results, a future replication across scanners would be a preferable advance.

The individual scan prediction of favorable versus non-favorable surgery outcome in male patients yielded a sensitivity of 100% with a specificity of 88% (Table 2). In terms of absolute numbers, our prediction correctly classified all but one male patient. This patient (M19 see Inline Supplementary Table S3) had post-surgical seizures up to two months after surgery and was thus determined to be ILAE class 3 at the time of the data analysis. However, we meanwhile contacted the physician in charge and noted that this patient has remained seizure free for more than the last two years. From the present point of view, this patient should now be assigned to the ILAE class 1. Hence, this patient is in a manner of speaking a ‘true misclassification’. In other words, the classifier actually chose the right category for this male patient. Finally, the man is remedied from his seizures by surgery.

The individual scan prediction of favorable versus non-favorable surgery outcome in male patients yielded a sensitivity of 100% with a specificity of 88% (Table 2). In terms of absolute numbers, our prediction correctly classified all but one male patient. This patient (M19 see Inline Supplementary Table S3) had post-surgical seizures up to two months after surgery and was thus determined to be ILAE class 3 at the time of the data analysis. However, we meanwhile contacted the physician in charge and noted that this patient has remained seizure free for more than the last two years. From the present point of view, this patient should now be assigned to the ILAE class 1. Hence, this patient is in a manner of speaking a ‘true misclassification’. In other words, the classifier actually chose the right category for this male patient. Finally, the man is remedied from his seizures by surgery.

The slightly larger female cohort totals 30 patients. The individual scan prediction of favorable and non-favorable surgery outcome in this cohort achieved a sensitivity of 100% with a specificity of 92%. That is, one woman in the Non-FO group was predicted incorrectly. Although this female patient (F22, see Inline Supplementary Table S4) has persistent post-surgical seizures, she is classified with a favorable surgery outcome. We reviewed all pre-surgical data of this patient and found conclusive interictal and ictal EEG findings. Furthermore, the post-surgical MRI demonstrated a complete resection of the left mTL structures with no visible complications. In summary, the reason for the post-surgical seizure persistence remains unclear. Nevertheless, she experienced a seizure reduction of more than 50% after surgery.

The slightly larger female cohort totals 30 patients. The individual scan prediction of favorable and non-favorable surgery outcome in this cohort achieved a sensitivity of 100% with a specificity of 92%. That is, one woman in the Non-FO group was predicted incorrectly. Although this female patient (F22, see Inline Supplementary Table S4) has persistent post-surgical seizures, she is classified with a favorable surgery outcome. We reviewed all pre-surgical data of this patient and found conclusive interictal and ictal EEG findings. Furthermore, the post-surgical MRI demonstrated a complete resection of the left mTL structures with no visible complications. In summary, the reason for the post-surgical seizure persistence remains unclear. Nevertheless, she experienced a seizure reduction of more than 50% after surgery.

### 4.2 No correlations of clinical characteristics and support vector weighting found

We analyzed the correlation of clinical characteristics such as the age at onset, the duration of the follow-up, the history of febrile seizures and seizure frequency with the support vector weighting given by the classifier. We found no correlations with any of these clinical characteristics (see Inline Supplementary Fig. S1). Furthermore, we found no differences in clinical characteristics of support vector machine support and non-support categorized patients (see Inline Supplementary Fig. S2). Here, the patient groups were closely matched and showed no significant difference in any clinical characteristic prior to classification (Table 1). Thus, they only differed in their surgery outcome, therewith proving once more the necessity of this method.

We analyzed the correlation of clinical characteristics such as the age at onset, the duration of the follow-up, the history of febrile seizures and seizure frequency with the support vector weighting given by the classifier. We found no correlations with any of these clinical characteristics (see Inline Supplementary Fig. S1). Furthermore, we found no differences in clinical characteristics of support vector machine support and non-support categorized patients (see Inline Supplementary Fig. S2). Here, the patient groups were closely matched and showed no significant difference in any clinical characteristic prior to classification (Table 1). Thus, they only differed in their surgery outcome, therewith proving once more the necessity of this method.

### 4.3 Neuroanatomical regions outside the margins of resection identified

As expected, pooling the male and female patients yielded no significant classification (balanced accuracy: 58%, p < 0.12). Moreover, the diversity of weighting distribution (Fig. 4) once more proves the necessity of separating the patients into a male and a female cohort. Overall, we found many extrahippocampal changes within the WM prior to surgery tending to be indicative of WM reorganization due to the seizures. Thus, considering the hippocampus in isolation as an evidence of a favorable surgery outcome seems to be insufficient. Notably, we found significantly more regions in the male patients associated with a favorable surgery outcome (p < 0.001; Chi-square test). Here, the men particularly exhibit disparities in brain regions contralateral to the seizure focus (p < 0.001; Chi-square test). Conversely, the female patients displayed a significant lateralization ipsilateral to their seizure focus (p < 0.001; Chi-square test) and possessed overall more areas associated with a non-favorable surgery outcome (p < 0.001; Chi-square test). Briefly, the structures involved in the patients' surgery outcome clearly differ between men and women. Thus, gender should be considered separately when predicting the individual surgery outcome of a patient.

Although a complete discussion of a possible pathology-specific reorganization within the WM in mTLE patients is far beyond the scope of this article, several key findings deserve to be mentioned. As obviously this was a highly selected population of left mTLE patients, these findings cannot be extrapolated to all mTLE patients. However, both cohorts yielded disparities in the SLF I. While the WM changes in male patients were associated with the FO group, the changes in women were predominantly involved with the Non-FO group. Differences in all aspects of the CB were found in both hemispheres for the male cohort. The contralateral capsule system was involved into prediction in both cohorts. The men appeared to have disparities in the ICA. However, the female patients showed differences in the extreme capsule. While we found disparities in the right posterior aspect of the ILF in male patients, the female patients showed differences in the posterior aspect of the ILF ipsilateral to their seizure focus. Brain regions apparently only different in the male patients were the FOF bilaterally, both caudate nuclei and the SLF III contralateral to the seizure focus. By contrast, the female patients showed WM changes of the SLF II in both hemispheres. The differences found in the contralateral temporal lobe in the female cohort comprised aspects of the MdLF. This abnormality was solely associated with the non-favorable surgery outcome (Focke et al., 2008). Regarding the lack of a robust structural basis given by the existing literature for the comparison of FO and Non-FO patients, we cannot refer to the consistency with our identified brain regions.

## 5 Conclusion

At present, the clinical diagnostic of mTLE patients is lacking a solid and reliable criterion for outcome prediction after selective amygdalohippocampectomy. We demonstrate the possibility to precisely predict this surgery outcome for left mTLE patients using their pre-surgical T_1_-weighted MR scans and support vector classification. Additionally, the identified gender-specific neuroanatomical findings of this work gave an insight into the reorganization of the WM prior to surgery. To this end, it merits further studies. Besides the straight forward investigation of right-sided mTLE patients, this method should be further extended to predict post-surgical outcome of specific mTLE subtypes such as MR-negative mTLE patients. In summary, a single T_1_-weighted MR scan in combination with our framework yields a strikingly robust and patient-specific pre-surgical prediction of a favorable or non-favorable surgery outcome. Since these MR scans are routinely acquired in clinical practice, the application of our method for a more reliable post-surgical outcome prediction can easily be incorporated into the pre-surgical workup. Hence, the pre-surgical workup of mTLE patients can be supported. It especially benefits from improved and above all individual patient information.

## Figures and Tables

**Table 1:** Summary of sociodemographic and clinical details of all patients. No significant difference in any clinical variable was observed. Abbreviations: FO = favorable outcome; IQR = interquartile range; Non-FO = non-favorable outcome; FU = follow-up; m = male cohort test FO versus Non-FO; f = female cohort test FO versus Non-FO; a = Mann–Whitney *U* test; b = four group test of male FO versus male Non-FO versus female FO versus female Non-FO; c = Kruskal–Wallis rank sum test; d = Fisher's exact test.

	Male cohort		Female cohort		Significance
	FO median (IQR)	Non-FO median (IQR)	FO median (IQR)	Non-FO median (IQR)	p-value
n	11	8	18	12	
Age at onset [in years]	18 (28)	13 (25)	14 (12)	12 (21)	0.89^m,a^; 0.8^f,a^; 0.97^b,c^
n had febrile seizures	4	4	5	4	0.66^m,d^; 0.71^f,d^; 0.75^b,d^
Pre-surgical seizure frequency [per month]	5 (6)	5 (3)	4 (5)	4 (5)	0.96^m,a^; 0.38^f,a^; 0.69^b,c^
Age at MRI [in years]	41 (21)	48 (6)	39 (15)	37 (28)	0.67^m,a^; 0.98^f,a^; 0.47^b,c^
Age at surgery [in years]	42 (21)	49 (6)	40 (15)	37 (28)	0.56^m,a^; 1^f,a^; 0.47^b,c^
FU [in months]	19 (8)	23 (17)	21 (17)	23 (22)	0.76^m,a^; 0.76^f,a^; 0.99^b,c^

**Table 2:** Summary statistics of the male and female classifiers evaluated in this study.

Statistics	Male cohort	Female cohort
Sensitivity	100%	100%
Specificity	88%	92%
Positive predictive value	92%	95%
Positive likelihood ratio	8	12
Area under the ROC curve	0.93	0.95
F-measure	0.96	0.97

## Appendix A Supplementary data

Supplementary Table 5 Summary statistics of the male and female classifier utilizing gray matter segments.
