# Supplementary material for: Prediction of post-surgical seizure outcome in left mesial temporal lobe epilepsy
Source: Neuroimage Clin. 2013 Jun 23;2:903–11. doi: 10.1016/j.nicl.2013.06.010 (PMC3778257; doi:10.1016/j.nicl.2013.06.010)
Supplement: Inline Supplementary Table S1 [file mmc1.docx]

**Supplementary Table 1**

Individual clinical details of all male patients

*Abbreviations:* MRI = magnetic resonance imaging; SPS = simple partial seizure; CPS = complex partial seizure; SGTCS = secondarily generalized tonic clonic seizure; ILAE = international league against epilepsy; AED = antiepileptic drugs; FU = follow-up; ZNS = zonisamide; LEV = levetiracetam; PGB = pregabalin; LTG = lamotrigine; OXC = oxcarbazepine; LCM = lacosamide; CLB = clobazam; CBZ = carbamazepine; PRM = primidone; ESL = eslicarbazepine; VPA = valproic acid; LRZ = lorazepam.

| **ID** | **Age at MRI** | **Age at onset** | **febrile seizures** | **Age at surgery** | **pre-surgical seizure type** | **seizure frequency per months** | **duration of follow up in months** | **last available outcome (ILAE)** | **AED at surgery** | **AED at last FU** | |
| --- | --- | --- | --- | --- | --- | --- | --- | --- | --- | --- | --- |
| M01 | 56 | 24 | 1 | 56 | CPS, SGTCS | 3 | 24 | 1 | ZNS | PGB |  |
| M02 | 51 | 33 | 0 | 51 | CPS, SGTCS | 3 | 24 | 1 | LEV, LTG | LEV, LTG |  |
| M03 | 53 | 1 | 0 | 52 | SPS, CPS, SGTCS | 6 | 18 | 1 | LTG. LEV | LEV |  |
| M04 | 32 | 13 | 1 | 32 | SPS, CPS | 4 | 16 | 2 | LEV, LTG | LEV, LTG |  |
| M05 | 41 | 5 | 0 | 42 | SPS, SGTCS | 3 | 30 | 1 | LEV, OXC | LEV, OXC |  |
| M06 | 57 | 34 | 0 | 57 | SPS, CPS, SGTCS | 20 | 13 | 1 | OXC | LTG |  |
| M07 | 50 | 26 | 0 | 50 | SPS, CPS | 5 | 26 | 1 | LEV, LTG | LEV, LTG |  |
| M08 | 22 | 1 | 0 | 23 | SPS, CPC | 12 | 23 | 1 | LEV, LCM | LEV, LCM |  |
| M09 | 31 | 3 | 1 | 31 | SPS, CPS | 1 | 19 | 2 | LTG | LTG |  |
| M10 | 27 | 18 | 1 | 28 | SPS, SGTCS | 5 | 12 | 1 | LEV, LTG | LEV, LTG |  |
| M11 | 37 | 35 | 0 | 37 | CPS, SGTCS | 10 | 19 | 1 | LEV, LTG | LEV, LTG |  |
| M12 | 67 | 46 | 0 | 67 | CPS, SGTCS | 40 | 12 | 4 | LEV, LTG, OXC | LEV, LTG, OXC |  |
| M13 | 47 | 5 | 1 | 47 | CPC, SGTCS | 4 | 45 | 3 | LEV, LTG, CLB | LTG, LEV |  |
| M14 | 21 | 18 | 0 | 21 | CPS | 4 | 12 | 3 | LTG, LEV | LTG, LEV |  |
| M15 | 49 | 15 | 1 | 50 | CPS | 1 | 30 | 3 | LEV, LTG, CLB | LEV, LTG |  |
| M16 | 46 | 3 | 1 | 46 | SPS, CPS | 8 | 28 | 4 | LEV | LEV |  |
| M17 | 44 | 11 | 0 | 45 | SPS, CPS | 3 | 12 | 4 | OXC | CBZ |  |
| M18 | 51 | 5 | 0 | 51 | SPS, CPS | 5 | 25 | 4 | LTG | LTG, OXC |  |
| M19 | 51 | 42 | 1 | 52 | SPS, CPS, SGTCS | 5 | 20 | 3 | LEV |  |  |
