# Supplementary material for: Prediction of post-surgical seizure outcome in left mesial temporal lobe epilepsy
Source: Neuroimage Clin. 2013 Jun 23;2:903–11. doi: 10.1016/j.nicl.2013.06.010 (PMC3778257; doi:10.1016/j.nicl.2013.06.010)
Supplement: Inline Supplementary Table S2 [file mmc2.docx]

**Supplementary Table 2**

Individual clinical details of all female patients

*Abbreviations:* MRI = magnetic resonance imaging; SPS = simple partial seizure; CPS = complex partial seizure; SGTCS = secondarily generalized tonic clonic seizure; ILAE = international league against epilepsy; AED = antiepileptic drugs; FU = follow-up; ZNS = zonisamide; LEV = levetiracetam; PGB = pregabalin; LTG = lamotrigine; OXC = oxcarbazepine; LCM = lacosamide; CLB = clobazam; CBZ = carbamazepine; PRM = primidone; ESL = eslicarbazepine; VPA = valproic acid; LRZ = lorazepam.

| **ID** | **Age at MRI** | **Age at onset** | **febrile seizures** | **Age at surgery** | **pre-surgical seizure type** | **seizure frequency per months** | **duration of follow up in months** | **last available outcome (ILAE)** | **AED at surgery** | **AED at last FU** |
| --- | --- | --- | --- | --- | --- | --- | --- | --- | --- | --- |
| F01 | 35 | 2 | 0 | 36 | SPS, CPS | 4 | 38 | 1 | LEV | LEV |
| F02 | 64 | 14 | 0 | 64 | CPS | 3 | 48 | 1 | LTG. LEV | PGB. LRZ |
| F03 | 22 | 0 | 0 | 23 | CPS | 30 | 25 | 1 | LEV, CLB, LTG | LTG |
| F04 | 49 | 2 | 1 | 49 | SPS, CPS | 1 | 39 | 1 | PRM, LEV | PRM, LEV |
| F05 | 39 | 29 | 0 | 39 | CPS, SGTCS | 3 | 36 | 1 | LEV, LTG | LTG |
| F06 | 44 | 15 | 0 | 44 | SPS, CPS | 4 | 13 | 1 | LEV | LEV, LCM |
| F07 | 45 | 21 | 0 | 46 | CPS | 15 | 24 | 1 | LEV, OXC | LEV, OXC |
| F08 | 42 | 9 | 0 | 42 | SPS, CPS | 60 | 28 | 1 | LTG, LEV | LEV, LTG |
| F09 | 27 | 13 | 1 | 27 | SPS, CPS | 2 | 12 | 1 | LTG | LTG |
| F10 | 30 | 18 | 1 | 31 | CPS, SGTCS | 5 | 12 | 1 | LEV, OXC | LEV, OXC |
| F11 | 33 | 13 | 0 | 34 | SPS, CPS, SGTCS | 2 | 24 | 2 | LEV, LTG | LEV, LTG |
| F12 | 45 | 3 | 1 | 46 | CPS | 3 | 12 | 1 | LEV | LEV |
| F13 | 48 | 30 | 0 | 49 | SPS, CPS, SGTCS | 15 | 12 | 1 | LTG, PGB | LTG, PGB |
| F14 | 22 | 13 | 0 | 22 | SPS, CPS | 4 | 29 | 1 | LTG, LCM | LTG |
| F15 | 32 | 29 | 0 | 32 | SPS | 8 | 18 | 2 | LEV, LCM | LEV, LCM |
| F16 | 54 | 14 | 0 | 54 | SPS, CPS, SGTCS | 1 | 12 | 1 | LEV, LTG | LEV, LTG |
| F17 | 39 | 36 | 0 | 40 | CPS | 4 | 17 | 2 | LTG | LTG |
| F18 | 29 | 15 | 1 | 29 | CPS | 6 | 12 | 2 | LEV | LEV |
| F19 | 24 | 6 | 0 | 26 | CPS | 1 | 38 | 3 | LTG | LTG |
| F20 | 25 | 11 | 1 | 25 | CPS, SGTCS | 5 | 54 | 3 | LTG, LEV | LTG |
| F21 | 33 | 32 | 0 | 33 | SPS, CPS, SGTCS | 10 | 52 | 3 | LEV, LTG, LRZ | LTG, LEV, LCM, LRZ |
| F22 | 32 | 6 | 1 | 32 | SPS, CPS, SGTCS | 7 | 34 | 4 | LEV, CLB | LCM, VPA, LTG, ZNS |
| F23 | 56 | 33 | 0 | 57 | SPS, CPS | 1 | 16 | 4 | LTG | LTG |
| F24 | 43 | 1 | 0 | 43 | CPS, SGTCS | 2 | 22 | 4 | CBZ, LTG | LTG, ESL |
| F25 | 23 | 13 | 0 | 24 | CPS, SGTCS | 1 | 23 | 4 | LTG | LTG |
| F26 | 26 | 9 | 0 | 26 | SPS, CPS | 4 | 12 | 3 | OXC | OXC, LEV |
| F27 | 50 | 15 | 1 | 51 | CPS, SGTCS | 6 | 23 | 4 | CBZ, CLB | CBZ |
| F28 | 70 | 34 | 0 | 72 | CPS | 6 | 12 | 3 | LEV, LCM | LTG |
| F29 | 41 | 1 | 1 | 41 | CPS, SGTCS | 3 | 16 | 4 | CBZ, LTG | LTG, OXC |
| F30 | 62 | 21 | 0 | 63 | SPS, CPS | 2 | 12 | 3 | LTG | LTG |
