# Supplementary material for: Prediction of post-surgical seizure outcome in left mesial temporal lobe epilepsy
Source: Neuroimage Clin. 2013 Jun 23;2:903–11. doi: 10.1016/j.nicl.2013.06.010 (PMC3778257; doi:10.1016/j.nicl.2013.06.010)
Supplement: Inline Supplementary Table S3 [file mmc3.docx]

**Supplementary Table 3**

Prediction of male patients with their individual subject results

| Subject Number | Actual diagnostic / outcome label  1-favorable  -1-non-favorable | SVM Predicted label^*^ |
| --- | --- | --- |
| M01 | 1 | 0.75914 |
| M02 | 1 | 0.075861 |
| M03 | 1 | 0.4679 |
| M04 | 1 | 0.059285 |
| M05 | 1 | 0.43343 |
| M06 | 1 | 0.67053 |
| M07 | 1 | 1.0851 |
| M08 | 1 | 0.73569 |
| M09 | 1 | 0.65359 |
| M10 | 1 | 0.19013 |
| M11 | 1 | 0.92097 |
| M12 | -1 | -1.2076 |
| M13 | -1 | -0.097875 |
| M14 | -1 | -0.60293 |
| M15 | -1 | -0.20462 |
| M16 | -1 | -0.59343 |
| M17 | -1 | -0.63184 |
| M18 | -1 | -0.023385 |
| M19 | -1 | 0.55178 |

Predicted label designated as ^*^>0=favorable or <0=non-favorable outcome. The further apart a prediction is from 0, the stronger is the evidence of that patient being in either of the groups.
