# Supplementary material for: Prediction of post-surgical seizure outcome in left mesial temporal lobe epilepsy
Source: Neuroimage Clin. 2013 Jun 23;2:903–11. doi: 10.1016/j.nicl.2013.06.010 (PMC3778257; doi:10.1016/j.nicl.2013.06.010)
Supplement: Inline Supplementary Table S4 [file mmc4.docx]

**Supplementary Table 4**

Prediction of female patients with their individual subject results

| Subject Number | Actual diagnostic / outcome label  1-favorable  -1-non-favorable | SVM Predicted label^*^ |
| --- | --- | --- |
| F01 | 1 | 0.69374 |
| F02 | 1 | 0.057686 |
| F03 | 1 | 0.61948 |
| F04 | 1 | 0.31212 |
| F05 | 1 | 0.54516 |
| F06 | 1 | 0.62866 |
| F07 | 1 | 0.78818 |
| F08 | 1 | 0.14086 |
| F09 | 1 | 0.3572 |
| F10 | 1 | 0.66793 |
| F11 | 1 | 1.2308 |
| F12 | 1 | 0.54714 |
| F13 | 1 | 0.52743 |
| F14 | 1 | 0.3758 |
| F15 | 1 | 1.9909 |
| F16 | 1 | 0.26292 |
| F17 | 1 | 0.95757 |
| F18 | 1 | 0.87285 |
| F19 | 1 | -0.46047 |
| F20 | -1 | -0.020884 |
| F21 | -1 | -0.18794 |
| F22 | -1 | 0.35073 |
| F23 | -1 | -0.1866 |
| F24 | -1 | -0.18786 |
| F25 | -1 | -0.085392 |
| F26 | -1 | -0.086466 |
| F27 | -1 | -0.29476 |
| F28 | -1 | -0.19536 |
| F29 | -1 | -0.3722 |
| F30 | -1 | -0.21038 |

Predicted label designated as ^*^>0=favorable or <0=non-favorable outcome. The further apart a prediction is from 0, the stronger is the evidence of that patient being in either of the groups.
