# Supplementary material for: Prediction of post-surgical seizure outcome in left mesial temporal lobe epilepsy
Source: Neuroimage Clin. 2013 Jun 23;2:903–11. doi: 10.1016/j.nicl.2013.06.010 (PMC3778257; doi:10.1016/j.nicl.2013.06.010)
Supplement: Supplementary Table 5 — Summary statistics of the male and female classifier utilizing gray matter segments. [file mmc5.docx]

**Supplementary Table 5**

Summary statistics of the male and female classifier utilizing gray matter segments.

| **Statistics** | **Male cohort** | **Female cohort** |
| --- | --- | --- |
| Sensitivity | 91% | 72% |
| Specificity | 50% | 67% |
| Positive predictive value | 71% | 76% |
| Positive likelihood ratio | 2 | 2 |
| Area under the ROC curve | 0.71 | 0.69 |
| F-measure | 0.8 | 0.74 |
